# Shifting Perspectives of Translational Research in Bio-Bactericides: Reviewing the *Bacillus amyloliquefaciens* Paradigm

**DOI:** 10.3390/biology10111202

**Published:** 2021-11-18

**Authors:** Anastasia Dimopoulou, Ioannis Theologidis, Adamantia Varympopi, Dimitris Papafotis, Glykeria Mermigka, Aliki Tzima, Nick J. Panopoulos, Nicholas Skandalis

**Affiliations:** 1 Institute of Molecular Biology and Biotechnology, FORTH, 70013 Heraklion, Greece; anastasia_dimopoulou@imbb.forth.gr (A.D.); glykeria_mermigka@imbb.forth.gr (G.M.); 2 Laboratory of Pesticides’ Toxicology, Benaki Phytopathological Institute, 14561 Athens, Greece; i.theologidis@bpi.gr; 3 Enzyme and Microbial Biotechnology Unit, Department of Biology, National and Kapodistrian University of Athens, 15784 Athens, Greece; avarympopi@biol.uoa.gr (A.V.); dpapafotis@biol.uoa.gr (D.P.); 4 Laboratory of Plant Pathology, Department of Crop Production, School of Agricultural Production Infrastructure and Environment, Faculty of Crop Science, Agricultural University of Athens, 11855 Athens, Greece; aliki@aua.gr; 5 Department of Environmental Science, Policy and Management, University of California, Berkeley, CA 94720, USA; npanopoul@gmail.com; 6 Health Sciences Campus, Keck School of Medicine, University of Southern California, 1441 Eastlake Ave, Los Angeles, CA 90033, USA

**Keywords:** biological control agents, BCAs, bacterial biological control, environmental impact

## Abstract

**Simple Summary:**

The continuous reduction of approved conventional microbicides, due to health concerns and the development of plant-pathogen resistance, has been urged for the use of safe alternatives in crop protection. Several beneficial bacterial species, termed biological control agents, are currently used in lieu of chemical pesticides. The approach to select such bacterial species and manufacture commercial products has been based on their biocontrol effect under optimal growth conditions, which is far from the real nutrient-limited field conditions of plant niches. It’s important to determine the complex interactions that occur among BCAs, plant host and niche microbiome to fully understand and exploit the potential of biological control agents. Furthermore, it’s crucial to acknowledge the environmental impact of their long-term use.

**Abstract:**

Bacterial biological control agents (BCAs) have been increasingly used against plant diseases. The traditional approach to manufacturing such commercial products was based on the selection of bacterial species able to produce secondary metabolites that inhibit mainly fungal growth in optimal media. Such species are required to be massively produced and sustain long-term self-storage. The endpoint of this pipeline is large-scale field tests in which BCAs are handled as any other pesticide. Despite recent knowledge of the importance of BCA-host-microbiome interactions to trigger plant defenses and allow colonization, holistic approaches to maximize their potential are still in their infancy. There is a gap in scientific knowledge between experiments in controlled conditions for optimal BCA and pathogen growth and the nutrient-limited field conditions in which they face niche microbiota competition. Moreover, BCAs are considered to be safe by competent authorities and the public, with no side effects to the environment; the OneHealth impact of their application is understudied. This review summarizes the state of the art in BCA research and how current knowledge and new biotechnological tools have impacted BCA development and application. Future challenges, such as their combinational use and ability to ameliorate plant stress are also discussed. Addressing such challenges would establish their long-term use as centerfold agricultural pesticides and plant growth promoters.

## 1. Introduction

Plant diseases caused by bacterial and fungal pathogens create major limitations on crop production with severe annual losses worldwide [1,2]. The consistent and efficient control of plant diseases may be extremely hard, facing pressure by consumer preferences for susceptible cultivars and changing environmental conditions [2]. New disease threats are emerging all over the world. Young grapevine decline caused by a complex of fungal poses a serious threat to the grapevine nursery and viticulture industry [3]. Diseases caused by bacterial pathogens, such as *Xyllela fastidiosa* and *Pseudomonas syringae* pv. *actinidiae*, which originated a few years ago in Italy and New Zealand, respectively [4,5], have already become epidemics. Citrus greening accounts for the loss of over 50% of Florida’s citrus plants and constitutes a global threat [6]. To respond to the growing list of pathogens, a pallet of local and systemic organic fungicides has been developed. In contrast, the already limited list of bactericides, mostly based on copper, is shortened due to concerns of residues in food and water and its environmental impact [7,8,9]. Biological control, the use of microbial antagonists termed as biological control agents (BCAs) to suppress diseases [10] gained ground in lieu of chemicals as a safe alternative for disease management [11]. Microbial BCAs are microorganisms such as bacteria, fungi, or viruses that attack specific plant pests. They affect pathogens with multiple modes of action which can be direct and/or indirect. The direct action is considered to result from competition for nutrients and environmental niches, antibiosis through secretion of harmful metabolites, and inhibition of pathogen colonization of the host plant. The indirect action includes induction of plant defenses and plant growth promotion [12].

This review aims to introduce BCAs to readers that are unfamiliar with crop protection and focus on the use of BCAs as bactericides. We focus on *B. amyloliquefaciens*, which dominates the market as a bio-bactericide. The scientific premise on its mode of action is analyzed and correlated to current approaches for the development of commercial formulations. Limitations of traditional methodologies are highlighted and the need for a holistic approach is suggested. Future challenges for researchers in agroindustry are analyzed, such as the need for improvement of active ingredients, the extension of their use, and environmental impacts. The scope of this article is to introduce BCAs to readers and highlight state-of-the-art research that has changed perspectives and their use. We used keyword research in popular databases such as Google Scholar, Scopus, and PubMed. The keywords used were BCAs, bacterial biocontrol agents, and environmental impact. Only articles published in English and from reputed journals from individual fields were considered. A sum of 196 publications was selected to follow the progress in the field across the globe over the past two decades.

## 2. An Overview of Bacterial Pesticides

### 2.1. The Global Market

Nowadays, it is crucial more than ever to protect our soils by reducing pesticides and agrochemicals by 30–50% in the next decade thus enhancing the health of every living organism including human life [13]. For this purpose, the use of biopesticides aims to replace pre- or post-harvest applications of conventional pesticides to eliminate their residues [14,15]. The global crop protection market offers several fungicidal products but a limited number of bactericidal compounds. The use of bacterium-based agents can provide a great source of microbicides.

Globally, the USA and Europe comprise the most extensive regional markets for biocontrol products [16]. In 2018, the global biofungicide market was estimated to be USD 1208.2 million and it is expected to reach USD 2877.2 million by 2024, as many big agrochemicals companies broaden their research and development (R&D) section towards biocontrol [17]. Most of the bacterial strains commercialized as biopesticides, fungicides, and bactericides belong to *Agrobacterium*, *Bacillus*, and *Pseudomonas* genera (Table 1). Unlike bioinsecticides, which have been commercialized since 1938 [18], the first biocontrol agent, *Agrobacterium radiobacter* strain K84, was registered in 1979 against crown gall [15]. Currently, half of the commercial products are based on *Bacillus* species (Table 1), because of their multiple ways of action and their spore stability [19] which guarantee long shelf life. Pseudomonads face registration obstacles due to formulation issues and the risk to act as opportunistic human pathogens. Nevertheless, Blightban A506 (*Pseudomonas fluorescens* A506) has been registered for use against *Erwinia amylovora*, the causing agent of fire blight disease [20].

In Europe, the revised version of Regulation (EC) No. 1107/2009 directs commercialization [21]. A two-tier registration system is imposed to evaluate BCAs according to requirements for the active substance (e.g., identification, mode of action, toxicity testing) and formulation. Additionally, BCAs should be listed as an active substance based on peer review by all EU member states, the European Food Safety Authority (EFSA), and the European Commission [21]. Products that await authorization from every EU member country should be accompanied by efficacy and safety certifying data over a 24-month period. The legislative authorities of each EU member state accredit institutions to oversee field trials in accordance with Good Experimental Practice [21]. Predictably, the expensive EU registration procedures discourage small and medium-sized companies. For example, *Pseudomonas chlororaphis* (Cedomon) was registered 10 years after its file submission for suppression of soil-borne pathogens of barley and wheat [22]. Additionally, this is the reason why many plant growth-promoting bacteria which may have biocontrol effects are registered in the EU as biofertilizers than biopesticides [23].

On the contrary, the registration procedure in the USA is simplified because the Environmental Protection Agency (EPA), which supervises this procedure, handles biocontrol products as safer than chemical compounds [24]. This assumption helps to complete registration in a minimum of 12–24 months (compared with 84 months in Europe). Moreover, certain health and environmental safety assessments performed in the USA are considered insufficient by EU legislators and that explains why most US products are unavailable in the EU market [25].

The vast majority of bacterial BCAs are registered against fungal diseases, and some extended their label as bactericides based on additional evidence. For example, *Bacillus amyloliquefaciens* MBI 600 was primarily registered as a pesticide active ingredient against *Aspergillus* spp., *Fusarium* spp., and *Rhizoctonia* spp. in 1994 [26]. However, the latest revised label of Serifel, which contains the same BCA, refers to additional antibacterial action towards *Erwinia* spp., *Pseudomonas* spp., and *Xanthomonas* spp. [27]. In support, a study of 215 patents showed that the majority of potential microbial pesticide organisms had a fungicidal mode of action, and only seven indicated bactericidal activity [28].

### 2.2. The Discovery of Bacterial Biopesticides

Over the past 120 years, research has repeatedly demonstrated that phylogenetically diverse microorganisms can act as natural antagonists of various plant pathogens [29]. The first commercial BCA against plant diseases, *Bacillus subtilis*, dates back to 1897 [30]. Notable examples of pioneer research include the first evidence of suppression of damping-off of pine seedlings [31] and potato scab disease [32] through the application of antagonistic fungi. Additionally, parasitism of the pathogen *Rhizoctonia solani* by *Trichoderma* (*Gliocladium*) virens, a well-known fungal BCA nowadays [33], and the use of *Agrobacterium radiobacter* and *Pseudomonas fluorescens* for prevention of crown gall on woody crops and fire blight in orchards, respectively [34,35].

In the late 1990s, competition assays led to the identification of several *Pseudomonas* and *Bacillus* species that were able to produce antibiotics and plant growth-promoting compounds, known as plant growth-promoting rhizobacteria (PGPRs) [36].

### 2.3. The Traditional Approach to Develop Bacterial Pesticides

The traditional approach to manufacturing a commercial BCA product is to isolate strains from disease-suppressive soils, test, produce industrially, preserve, store, and formulate them [37]. Efficacy evaluation of such microorganisms is mostly based on the secretion of a broad spectrum of secondary metabolites with antimicrobial activity [11]. In vitro testing of biocontrol activity is biased in favor of antibiosis biocontrol but excludes the contribution of other modes of action. In the two past decades, this approach has been evolved and additional criteria were proposed based on the multiple modes of action of biocontrol agents including competition for nutrients and space, successful colonization under different environmental conditions, and stimulation of plant-induced resistance (ISR) [38,39,40]. Adhesion to plant roots and formation of biofilm, both being prerequisites for colonization and establishing relationships with host plants, are also taken into account [37]. Biofilm is a self-produced extracellular matrix [41] that allows the exchange of nutrients, toxins, and protection from environmental stresses and antimicrobial compounds [42].

**Figure 1:**
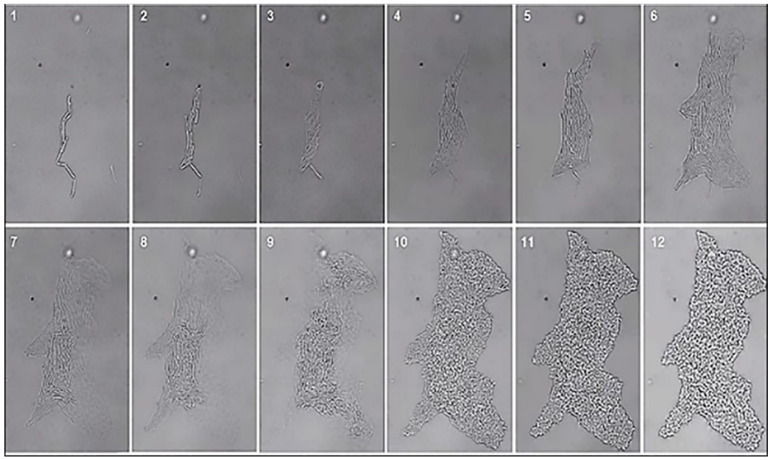
*B. amyloliquefaciens* strain MBI600 (the active ingredient of Serifel, BASF SE) spontaneously forms biofilms. Based on a previously described method [43], MBI600 single cells were observed in LB agar medium using a Leica SP8 time-lapse confocal microscope, over a 9 h period.

Most bacteria grow planktonically in vitro and solid minimal media are needed to visualize their innate ability to form biofilms. *B. amyloliquefaciens* strain MBI600, however, has an innate ability to spontaneously form biofilms; it can form biofilms in media that favor planktonic growth. We observed, through Leica SP8 time-lapse confocal microscope, that single MBI600 cells became sessile, duplicated, and organized in chains Figure 1 (1–3) (Video S1). Then, they adhered to each other and formed a colony with guard cells at its edges facilitating a coordinated expansion Figure 1 (4–6). Three to four h later, cells emerged on a second layer Figure 1 (7–9), growth was suspended on the base of the biofilm which gained height and formed a matrix Figure 1 (10–12) (Video S1). This contrasts with (other) *Bacillus* spp. growth in planktonic media where colonies primarily expand in width to cover the available nutrient medium.

## 3. Shifting Perspectives in BCA Mode of Action and Application

The success of biological control involves a deep understanding of the modes of action of the antagonist, its interactions with the plant and the pathogen, and the mode and dose of application as well [44]. An important parameter affecting many aspects of the biocontrol effect is the formulation of BCAs [15]. The biocontrol performance relies also on the colonization of the plant surface by biocontrol agents on specific physiological stages of the plant in order to develop a sufficiently high level of resistance [45]. For example, experimental results suggest that optimum fire blight control can be achieved by applying *Pseudomonas fluorescens* A506 during the main bloom with repetitive applications at 7- to 10-day intervals to achieve colonization of delayed blossoms [46]. Moreover, it can be observed from Table 1 that every formulation is designed for a specific application according to registration files. As with any biological system, three factors that influence attainment are water, food, and the environment. Water activity affects crucially the survival of biocontrol agents in formulations. However, a dry product is more favorable than other formulations, due to less weight to ship and the significantly lower risk of contamination [47].

### 3.1. “Let Them Eat Cake”: Antibiotic Production Is Conditional

Antibiotics are low-molecular-mass products of secondary metabolism, secreted by bacterial BCAs to compete with other bacteria for nutrients and space in an environmental niche (Figure 1). Such compounds exhibit antimicrobial activity by inhibiting pathogen growth at low concentrations. Two genera, *Pseudomonas* and *Bacillus*, have been well studied for the production of such antibiotics and their impact on disease management [48,49,50]. The most well-known antibiotic, pyrrolnitrin, is naturally produced by *Pseudomonas* spp.; after being processed, the fungicide fludioxonil can be derived [51]. Several other antibiotic compounds such as pyoluteorin, hydrogen cyanide, phloroglucinols, and cyclic lipopeptides have been characterized as being produced by Pseudomonads [52]. Pyoluteorin and 2,4 diacetylphloroglucinol (2,4 DAPG) are antibiotics produced by *P. fluorescens* strains that have a strong inhibitory effect against the phytopathogen *Xanthomonas oryzae* pv. *oryzae* [53]. On the other hand, *Bacillus* spp. seem to exhibit the strongest inhibitory effect against a broader range of phytopathogenic bacteria due to the plethora of the produced antimicrobial compounds [54]. This antimicrobial spectrum includes (i) ribosomal peptides, bacteriocins, which inhibit the growth of Gram-positive bacteria such as *Bacillus* spp., *Clostridium* spp. and *Staphylococcus* sp. [55,56] or Gram-negative bacteria such as *Agrobacterium tumefaciens* [57], (ii) polyketides (PKS) with an antibacterial effect against *Erwinia amylovora*, *Xanthomonas oryzae* and *Ralstonia solanacearum* [58,59], (iii) non-ribosomal peptides and lipopeptides (NRPs and LPs) with strong antifungal activity apart from surfactins which have been shown to inhibit pathogenic bacteria such as *R. solanacearum* and *Xanthomonas* spp [60,61].

The production of antibiotics is regulated by various environmental factors such as carbon sources, temperature, pH, and oxygen availability [62,63] (Figure 1). For example, high temperatures (>37 °C) favor the production of surfactins while low temperatures that of fengycins and iturins [64]. Additionally, the depletion of carbon, nitrogen, phosphate, iron, or other nutrient sources can trigger the secondary metabolism and thus the production of antibiotics. Indeed, Duffy and Défago [65] have shown that glucose was able to stimulate antibiotic production in almost all *Pseudomonas* strains, while phosphate repressed it. Similar findings of phosphate were observed for kanosamine production of *Bacillus cereus* [66].

In current biocontrol efficacy evaluation tests in vitro, pathogen susceptibility against secondary metabolites with antibiotic function is often assessed on nutrient media in which microbial antagonists coexist in dual cultures. Alternatively, targeted microorganisms are grown either in the presence of the culture supernatant of a particular BCA or the purified concentration of the metabolite. Although these approaches have many advantages, one main disadvantage is that the production of antimicrobial metabolites depends on the nutrient concentration of the chosen medium. According to Lugtenberg et al. [67], nutrient media that are being used in these bioassays are 100 times richer in nutrients than rhizosphere, thus quantities of secondary metabolites are higher at in vitro systems compared to natural habitats.

**Figure 2:**
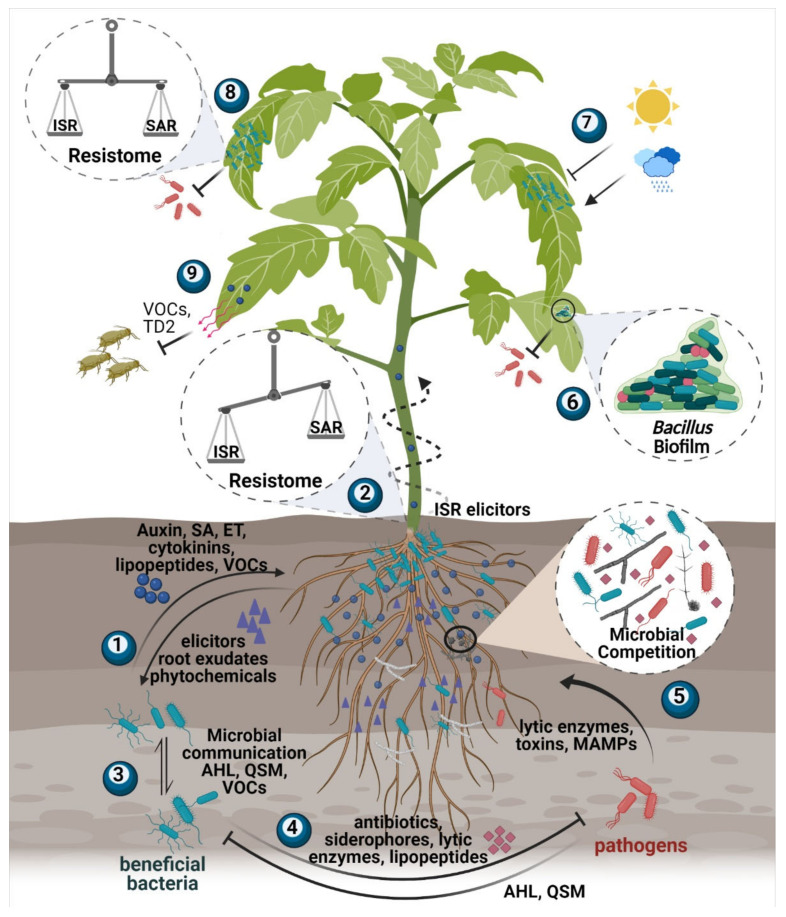
A schematic representation of the multifaceted interactions among BCAs, host plants, pathogens, and plant microbiota. Following rhizosphere application, BCAs and other beneficial bacteria perceive host signals and are recruited by the plant host (**1**). BCAs colonize roots and provide metabolites to plant roots that support growth and activate signaling pathways that trigger the Induced Systemic Resistance (ISR) and suppress Systemic Acquired Resistance (SAR) signaling (**2**). In parallel, BCAs interact with rhizosphere microbiota. This involves inter- or intra-specific communication by secretion of quorum sensing molecules (**3**) or antagonism with commensal bacteria and plant pathogens. Antagonism involves antibiosis by the production of bactericidal metabolites (**4**) and competition for nutrients and space (**5**). BCA colonization of the plant is largely dependent on its ability to form biofilm on roots but especially on leaves and crops in which the epidermis is a nutrient-limited hostile environment (**6**). Colonization of aerial parts largely depends on environmental conditions (**7**), humidity being the dominant factor. Following leaf colonization, microbial elicitors are perceived by plant receptors and trigger synergistically ISR and SAR (**8**) but also defense responses to insect pests by the production of volatile compounds of insecticidal enzymes (**9**). SA, Salicylic Acis; ET, Ethylene; VOCs, Volatile Organic Compounds; AHLs, N-Acyl homoserine lactone; QSM, Quorum Sensing Molecules; MAMPs; Microbe-Associated Molecular Patterns; ISR, Induced Systemic resistance; SAR, Systemic Acquired Resistance; TD2, Threonine dehydratase 2 biosynthetic protein. This illustration was created using the BioRender online software (https://biorender.com/ accessed on 21 January 2020).

In parallel with advances regarding the abiotic conditions and nutrient effect on antibiotic production by BCAs, research also has focused on respective effects by interspecific competition [68,69,70] (Figure 2). Because of the importance of biotic and abiotic factors that make antibiotic production conditional, new techniques are needed to optimize the evaluation of the actual amount of bacterial metabolites produced in situ compared to in vitro experiments [71,72].

### 3.2. The Role of Siderophores in Biocontrol

In support of the fact that major antimicrobial metabolites might not be produced when a BCA grows planktonically in axenic cultures, our group recently observed that the pallet of antimicrobial metabolites is extended under nutrient starvation [73]. In specific, they showed that production of the siderophore bacillibactin by *B. amyloliquefaciens* MBI 600, under iron limiting conditions, restrained in vitro and in planta growth of non-susceptible bacterial and fungal pathogens.

Siderophores are small non-ribosomal peptides secreted by bacteria, fungi, and plants. They function as high-affinity ferric chelators which solubilize iron before transport facilitate iron absorption and storage in iron-deprived niches [74,75]. Bacteria produce hydroxamate, catecholate, and in a few cases carboxylate types of siderophores, while fungi produce mainly hydroxamate and carboxylate types of siderophores [76].

Siderophores provide a selective advantage over microbial competitors [77] but also facilitate plant-microbe interactions [78] and are important for pathogen virulence [79]. In fact, microbes utilize siderophores produced by other microorganisms and are therefore referred to as xenosiderophores [80,81]. Even if the importance of siderophores for bacterial competition has been suggested decades ago [82], they have not been exploited in agriculture. Innovative medical uses of siderophores, such as the selective mediation of antibiotics to resistant clinical bacteria (Trojan horse strategy) [83] could be adopted in crop protection.

### 3.3. Reciprocal Perception Enables Host-Microbe Interactions

Competitive host colonization is crucial for a BCA to protect against diseases and to interact with the plant as a PGPR. Their ability to occupy space on plant rhizosphere and phyllosphere depends on biotic and abiotic parameters such as the host species, soil type, nutrient competitors, niche microbiota, pH, drought, salinity, etc. [84,85,86,87]. Plant roots exert considerable control over the composition of the rhizomicrobiome through the release of a wide range of chemoattractants and repellents including sugars, polysaccharides, amino acids, aromatic acids, aliphatic acids, fatty acids, sterols, phenolics, enzymes, proteins, plant growth regulators and secondary metabolites [88,89,90] (Figure 2). For example, precursors of plant phytohormones such as tryptophan (for indole-3-acetic acid) and aminocyclopropane-1-carboxylic acid (ACC) (for ethylene) are concentrated in the root tip region [91,92] and attract PGPR that uses them for the biosynthesis of phytohormones reviewed in [85]. PGPR-derived auxins (e.g., IAA) trigger physiological responses that induce defense induction [93] and activate auxin-responsive genes that enhance plant growth and increase their biomass [94].

**Figure 3:**
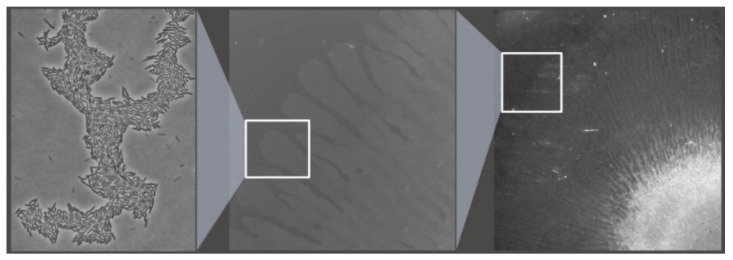
A multicellular *B. amyloliquefaciens* MBI600 community forms a biofilm in (M9) minimal media (A) but exhibits swarming motility when a synthetic analog of the root exudate malic acid (Merck) is added to the medium.

*B. amyloliquefaciens* strains have been shown to efficiently colonize the roots of *Arabidopsis thaliana* [95], *Lactuca sativa* [96], and other plants and overcome the antibacterial action of some plant root exudates [97] and perceive organic acids (malic and citric), polyamines (spermine) and other root exudates by methyl-accepting chemotaxis proteins in a kinase D mediated pathway to biosynthesize components and activate flagella and swarming movement while reducing biofilm formation [98]. Swarming is an adaptation of their locomotion machinery to achieve a specialized form of flagellum-driven motility in solid surfaces [99], mediated by the production of lipopeptide bio-surfactants that lower the surface thus facilitating movement [42] (Figure 3).

In contrast to the rhizosphere, the phyllosphere is an extreme and unstable habitat on which bacterial communities face acute fluctuations in temperature, humidity, and UV light irradiation and face limited access to nutrients [100,101] (Figure 2). Several studies are exhibiting that phyllosphere colonization by bacterial communities helps promote the wellness of the plant in many ways such as biocontrol, plant growth promotion, bioremediation of harmful chemicals, etc. [102]. In contrast to plant-pathogen leaf interactions [103], information on BCA-host leaf interactions and the reciprocal perception of signals is understudied.

### 3.4. BCAs Trigger Multifaceted Defense Responses

Successful BCA colonization results in plants exhibiting alterations of the biosynthetic and signaling pathways of phytohormones, activating components of the oxidative burst mechanism, and producing secondary metabolites that trigger defense responses in the presence of pathogens [104,105] or prime plants against potential pathogen attack [106]. Such responses are thought to be mediated by Jasmonic acid (JA) and Ethylene (ET) in an NPR1-dependent signaling pathway that triggers the induced systemic resistance (ISR) while suppressing salicylic acid (SA) levels and downstream signaling that induces the systemic acquired resistance (SAR) [107,108,109]. In support, *Bacillus amyloliquefaciens* FZB42 was able to enhance the expression of defense marker genes such as *pr1* (SA marker gene) and *pdf1.2* (JA/ET marker gene) in lettuce plants, while the co-existence of FZB42 and the lettuce pathogen *Rhizoctonia solani* activated only *pdf1.2* expression levels compared to *pr1* which expression levels were lower than the control FZB42 [110].

Our group recently found that *B. amyloliquefaciens* MBI 600 interacts with its tomato host by triggering a signaling network that differentially induced defense signaling pathways depending on plant part and dose of application [111] (Figure 2). In specific, the suggested dosage of the commercial formulation of MBI 600 (Serifel) induced defense by mediating synergistic cross-talk between JA/ET and SA-signaling. Low dosage primed plant defense by activation of SA-responsive genes, which reduced up to 80% the incidence of *Tomato spotted wilt virus* and delayed *Potato virus Y* systemic accumulation [112].

Diverse genera of BCAs have been reported to trigger ISR through the production of volatile compounds (2,3-butanediol) and lipopeptides (surfactins and fengycins) [113] causing significant reductions in the severity of various bacterial diseases in a diversity of host plants [114]. For instance, *B. amyloliquefaciens* IN937a secreted volatiles which triggered ISR in *Arabidopsis* seedlings challenged with the pathogen *Erwinia carotovora* subsp. *carotovora* cause of the soft rot disease [113]. Similar findings were reported for *Bacillus subtilis* QST 713 which successfully reduced disease severity of bacterial speck (*Pseudomonas syringae* pv. *tomato*) on tomato plants by triggering defense-related genes [115]. Additionally, Raaijmakers et al. [116] reported that circular lipopeptides surfactin and fengycin were capable of eliciting ISR in tomato plants and beans. Recently, a synergistic action of multiple secreted elicitors was detected in *B. amyloliquefaciens* SQR9 that induced ISR against the bacterial pathogen *Pseudomonas syringae* pv. *tomato* DC3000 through different signaling pathway genes [117].

The significant progress in the understanding of BCA-mediated induction of defense responses has allowed this area of research to turn from basic to translational. Nowadays BCAs are thought to indirectly affect non-target pests, such as viruses and insects. Advances in the usage of BCAs against plant viruses have been recently reviewed [118]. Leaf colonization by BCAs has been reported to induce JA-mediated resistance to herbivorous insects [119]. The colonization of plant roots by the rhizobacterium *Pseudomonas simiae* WCS417r elicits higher expression of the JA/ET dependent ORA59-branch than the JA-dependent MYC2 branch and triggers ISR against leaf-chewing insects [120]. In support, root colonization of cotton plants by *Bacillus* sp. induces JA levels rendering resistance against the herbivore *Spodoptera exigua* [121]. Additionally, colonization of tomato plants by mycorrhiza can prime systemic defense responses against insect attacks with increased expression of defense-associated genes allene oxide cyclase (*aoc*), *loxD* and protease inhibitors (PI-I, PI-II) [122]. *B. amyloliquefaciens* MBI600 have been reported to trigger ISR against insects through the expression of the JA-dependent MYC2 branch [111].

### 3.5. The Environmental Impact of Bio-Microbicides

Despite the effective control of plant diseases, chemical microbicides form a hazard for the environment due to contamination of surface and groundwater [123,124], soil [125,126,127], vegetation, and non-target organisms [128,129]. Moreover, they pose a serious risk to human health [130,131,132]. BCAs were introduced as an alternative crop protection method with minimum risk to the environment and human health. However, is this the case?

The massive release of BCAs has been hypothesized to have an impact on the plant microbiome. Low-throughput methods at first (plating, DGGE, FISH, Sanger sequencing) and high-throughput methods recently (next-generation sequencing) have analyzed the rhizosphere, and secondarily the phyllosphere microbiota following BCA application [133,134]. Overall, it appears that bacterial BCAs have a minor and transient effect on the microbiome after soil application [135,136,137,138,139,140], independently of the soil type and properties [141,142,143,144,145]. Interestingly, a study by Erlacher et al. [40] suggested that the plant microbiota shifted due to pathogen attack by *Rhizoctonia solani*, but *B. amyloliquefaciens* FZB42 reduced that effect. Qin et al. [146] also reported that interactions between BCAs and the microbial community might be beneficial for the plants in terms of controlling the bacterial wildfire disease.

The large-scale application of BCAs is recent and sporadic. Long-term effects of continuous application of these dominant environmental species in commensal bacteria, insect, and animal microbiota have not been studied yet and animal models are currently used to be assessed for acute toxicity rather than indirect effects [147].

### 3.6. The Multidisciplinary Approach to Study BCAs

BCA strains have been selected to be used in commercial microbicide formulations based on in vitro sensitivity tests and field trials [148].

Even if competitive host colonization is necessary for success in field trials, it has been understudied, and assumed rather than proved. Rhizosphere studies focus on the functional characterization of genes involved in biofilm formation [149,150] and monitor colonization based on microscopy, rather than actual counts of populations [151]. In support, *B amyloliquefaciens* MBI600 was found to successfully colonize primary roots of cucumber plants depending on the growth substrate of the roots [152]. Additionally, they have been based so far on artificial planting systems. Such systems utilize sterile substrates and hydroponics to eliminate rhizosphere microbiota and thus background bacteria in population counts. Unavoidably, they overlook niche competition that occurs in soil. Colonization of BCA in the phyllosphere is challenging due to the low availability of nutrients and organic matter on plant leaves. Although there is a scarcity of information about the colonization of BCAs in the phyllosphere [97], recent studies are indicating their abundance in the phyllosphere microbiota [153,154]. Wei et al. [145] have shown that *B. subtilis* was not only capable of maintaining on the leaves of strawberry for 8 days after application but also of increasing its abundance on new leaves. Colonization studies of BCA, other than *Bacillus* sp., have been sporadically reported on flower blossoms [45,155].

The low cost and massive sequencing of bacterial species [156,157] might reverse the procedure to develop BCAs. Identification of dominant environmental species by microbiota profiling of the rhizosphere and phyllosphere of economically important plant species allows us to rapidly identify numerous BCA candidates that successfully colonize plants, and colonization tests can be targeted and specific to confirm such potential. Whole-genome [152] and RNA sequencing [158], pathway analysis [159], and metabolomic analysis [160,161] is a fast way to identify the production of secondary metabolite microbicidal function under real environmental conditions [162] rather than optimal in vitro growth.

As aforementioned, BCA application is thought to affect herbivory by modification of plant signaling [163]. Plant and insect microbiome studies now supported an additional, indirect, effect to herbivory: BCA application might have a protective effect against the compositional shifts in leaf microbiota caused by herbivory, causing dysbiosis and increasing susceptibility to secondary bacterial infection [164]. BCAs are not, however, expected to directly affect herbivory. Insect bacteriocytes, haemolymph, gut, and salivary gland microbiota present low diversity, dominated by species (mostly *Enterobacteriaceae*) [165] different from those colonizing plants. It is therefore unlikely that shifts in plant microbiota by BCA application will lead to indirect effects to insects by the acquisition of microbial communities or genetic exchanges [166].

## 4. Future Challenges

The role of beneficial microorganisms is gaining importance in stress management and the development of climate change resilient agriculture. *B. amyloliquefaciens* has been found to increase abiotic stress tolerance [167] by accumulating compatible osmolytes such as proline, soluble sugars, etc. that help plants to maintain osmotic turgor [168] and thus alleviate salt tolerance [169]. For example, *B. amyloliquefaciens* SQR9 had enhanced salt stress tolerance of *Arabidopsis thaliana* and maize seedlings compared with the untreated samples through the increased total soluble sugar content leading to decreased cell death and enhanced peroxidase/catalase activity [170]. Additionally, the resistance of soybean under water-deficit conditions post application of thuricin 17 was observed due to the modifications of root structure, the increased root biomass and length, and the induced ABA and total nitrogen content [171]. All this is not of surprise, given the fact that *Bacillus* spp. dominate plant microbiomes under conditions of high temperature, salinity, and drought such as in Egypt [172].

*Pseudomonas putida* MTCC5279 also ameliorated drought stress in chickpea plants by modulating membrane integrity, osmolyte accumulation (proline, glycine betaine), and ROS scavenging ability. Similarly, PGPR might also help plants cope with flooding stress. Treatment of rice seedlings with the ACC deaminase-producing *Pseudomonas fluorescens* REN1 increased root elongation under constantly flooded conditions and salt stress effects were deteriorated [173]. *Variovorax paradoxus* 5C-2, an ACC deaminase-producing PGPR, enhanced salt tolerance, increased antioxidant enzyme activities, and upregulated ROS pathway genes of okra (*Abelmoschus esculentus* L.) plants under salinity stress [174]. A gibberellin-producing PGPR, *Serratia nematodiphila* increased pepper growth under low-temperature stress conditions. The inoculated pepper plants showed higher levels of GA and ABA but lower for salicylate and jasmonate [175]. Inoculation with *Burkholderia phytofirmans* PsJN modulated carbohydrate metabolism to reduce chilling damage to grapevine [176].

Numerous studies have been reported that combinational use of BCAs has improved the consistency of biocontrol across sites with variable conditions. Successful biocontrol using mixtures of BCAs has been tested against late blight in potato [177] cucumber, chili, and poplar diseases [178,179,180,181]. BCA combinations do not always provide increased control, as an antagonism between the BCAs may occur. For example, mixtures of *Pseudomonas fluorescens* A506, *Pantoea vagus* C9–1, and *Pantoea agglomerans* Eh252 were less effective than individual strains against *Erwinia amylovora* infection of pear blossoms, because peptide antibiotics of *Pantoea* spp. were degraded by an extracellular protease of *P. fluorescens* A506 [35]. A meta-analysis of experimental studies evaluating the combinational use of BCAs revealed that 10 out of 465 treatments indicated synergistic interactions, 70 cases resulted in improved efficacy, and 64 cases with significant antagonism [182].

Combining bioremediation with plant growth promotion could be a beneficial approach. A mixture of *B. amyloliquefaciens* with the fungal strain *Trichoderma virens* improved yields of corn and tomato, among other crops [183], and is commercially available. Combinations of *Trichoderma* sp. with *Bradyrhizobium* sp., for improved growth of soybean, and arbuscular mycorrhizal fungi and *Trichoderma harzianum* for improved growth and soilborne pathogen control are also commercially available [184,185]. Early attempts to utilize bacterial consortia had inconsistent effects on crop yield [186]. Recently, however, many studies are indicating the contribution of BCAs consortia to plant growth promotion [187,188].

A merging field of discovering new biocontrol agents is endophytes [189]. Endophytic bacteria confer plant growth promotion and suppression of biotic and abiotic stresses [190]. Important questions remain unanswered about the use of endophyte BCAs in crop production. Even if there is an urge to classify microbes as endophytic and develop them as BCAs, this qualification may be altered if they are found to cause disease in some plant species [191]. At present, there is a knowledge gap about genome differentiation between bacterial endophytes and plant pathogens [192]. Moreover, they may be passively absorbed by roots, since there is limited evidence of internally colonizing host species [37]. Additionally, endophytic inoculations are often unsuccessful in field experiments due to establishment problems [193]. Developing inocula containing highly effective microbes with a long shelf-life and high rhizosphere colonization rate poses a major challenge for commercialization.

Furthermore, technological developments in biotechnology open new possibilities in biocontrol such as genetic modification [194]. Despite all efforts, no genetically modified microorganisms (GMMs) with action are registered in the European Union (EU) yet, and only a few in the rest global market [195].

## 5. Conclusions

Despite the discovery of their pesticide function several decades ago, biological control agents practically were developed as biopesticides during the past decade. This was largely due to the poor understanding of their biology and interaction with hosts and the traditional approach to their development. The specialization of microbiologists in BCAs and biotechnology tools helped us understand their true potential. Scientists and the agroindustry shift, hesitantly, their focus from the production of secondary metabolites, which is conditional, to the exploitation of the triple BCA-host-microbe interaction in order to fully exploit BCAs for addressing crop production issues, including but not limited to disease protection.

## Figures and Tables

**Table 1:** Bacterial Biocontrol Agents (BCAs), currently registered as bactericides and fungicides in the European and American market. Target pathogens and plant hosts are listed based on label claims. Different colors in the target crop column indicate authorized use as bactericides (Bact), fungicides (F), or both (Both).

Bacterial BCAs	Brand Name	Manufacturer	Formulation	Application	Registration		Target Fungal Diseases	Target Bacterial Diseases	Crops	Authorizations
					Use	Region				
*Agrobacterium radiobacter* K84	Galltrol-A	AgBioChem	Cell culture	Cuts, roots, seeds and stems application	Bactericide	US		Crown gall (*Agrobacterium* *tumefaciens)*	Bact: Non-food bearing stone fruit and nut trees	US:40230-1
	Gallex		Flowable emulsion	Gals and cuts application				Crown gall (*Agrobacterium tumefaciens*), olive knot (*Pseudomonas savastanoi)*	Bact: Oleander, olive, ornamentals, stone and nut fruit trees	US:40230-2
*Agrobacterium radiobacter (syn.* strain K1062	Nogall	Basf	Cells in peat carrier (wettable powder)	Cuts, roots, seeds and stems application	Bactericide	US		Crown gall (*Agrobacterium Tumefaciens)*	Bact: Non-food bearing stone fruit and nut trees, ornamentals	US:62388-1
*Bacillus amyloliquefaciens* D747	Double Nickel 55	Certis USA L.L.C	Bacterial endospores (wettable powder)	Chemigation, cut/root, foliar and ground application	Bactericide, fungicide	EU	Blasts, blights, blotches, damping-off diseases, downy mildews, drops, eutypa, flyspecks, leaf curls, melanoses, molds, mummy berry, phomopsis, powdery mildews, rots, rusts, scabs, scurfs, shanks, shot hole, sigatoka, smuts, spots, vine decline	Blights, cankers, specks and spots (*Erwinia* spp. *Pseudomonas spp., Xanthomonas* spp.)	Both: Citrus, pome and stone fruit trees, coffee, herbs and spices, nut trees, tropical fruits, vegetables F:Berries, cereal grains, grapes, oilseed crops, pomegranates, sugar beets and tobacco	US: 70051-108
	Amylo-X			Foliar application		US	Blights, downy mildews, molds, powdery mildews, rots, sclerotinia	Fire blight (*Erwinia amylovora*), kiwi canker (*Pseudomonas syringae* pv. *actinidiae*)	Bact: Kiwifruit pome F: Berries grapes, mushrooms, and stone fruit trees, vegetables	EU: Reg. No 1316/2014 (Dossier complete (2011/253/EU)
*Bacillus amyloliquefaciens* strain FZB24	Taergo 2	Novozymes	Bacterial endospores (wettable powder)	Cut/root, foliar and ground application, ground incorporation	Bactericide, fungicide	US	Damping off diseases, blights, downy mildews, molds, powdery mildews, sclerotinia, spots	Specks (*Pseudomonas spp.*), spots (*Xanthomonas* spp.)	Both: Vegetables F: Ornamentals	US:70127-12
	Taergo			Foliar application		EU	Blights, damping off diseases, downy mildews, molds, powdery mildews, rots, spots	Specks (*Pseudomonas* spp.)	Both: vegetables F: Berries, grapes,	EU: Reg. (EU) 2017/806
*Bacillus amyloliquefaciens (subtilis)* MBI 600	Serifel	Basf	Bacterial endospores (wettable power	Foliar and ground application	Bactericide, fungicide	US	Anthracnose, blights, blotches, damping off diseases, diebacks, dots, downy mildews, drops, esca, eutypa, flyspeck, molds, mummy berry, phomopsis, powdery mildews, rots, rusts, scabs, shanks, scorches, scurfs, shot hole, spots	Aerial stem rot (*Erwinia carotovora*) cankers, specks and spots (*Pseudomonas* spp., *Xanthomonas* spp.), fire blight (*Erwinia amylovora*), walnut blight (*Xanthomonas campestris*	Both: Cereal grains, grapes, herbs and spices, oilseed crops, soybean, tobacco, sugar beets berries citrus, pome and stone fruit trees, tree nuts, vegetables	US:71840-18
				Foliar application	Fungicide	EU	Molds, sclerotinia		F: Berries, herbs and spices, grapes	EU: Reg. (EU) 2016/1429Reg. (EU) 540/2011
	Histick N/T (Beans, peanut, soybean)		Bacterial endospores (wettable power	Seed application	Fungicide	US	Damping off diseases		F: Beans, peanut, soybean	US: 71840-2
	Integral		Aqueous suspension	Ground application	Fungicide	US	Damping off diseases		F: Peanut, soybean	US: 71840-5
	Subtilex		Bacterial endospores (wettable powder)	Foliar and ground application	Fungicide	US	Damping off diseases, downy mildews, molds, powdery mildews		F: Bedding plants, ornamentals, tropical plants, vegetables	US: 71840-8
*Bacillus amyloliquefaciens (subtilis)* strain QST 713	Cease	Bioworks	Aqueous suspension	Aerial and ground application, chemigation	Bactericide, fungicide	US	Anthracose, blights, downy mildews, molds, powedery mildews, rots, rusts and sclerotinia	Blight (*Pseudomonas syringae*), fruit blotch (*Acidovorax avenae*), rots, specks and spots (*Erwinia* spp, *Pseudomonas* spp., *Xanthomonas* spp.)	Both: Berries, herbs and vegetables	US:264-1155-68539
	Rhapsody	Bayer Crop science	Concentrated cell suspension	Chemigation, foliar and ground application	Bactericide, fungicide	US	Anthracnose, brown patch, damping off diseases, gray mold, spots, powdery mildews, red thread, rust, septoria	Soft rot (*Erwinia* spp.), spots (*Erwinia* spp., *Pseudomonas* spp., *Xanthomonas* spp.)	Both: Landscape plants F: Berries, citrus, pome and stone fruit trees, golf courses lawns, turfs	US:264-1155
	Serenade Aso		Concentrated cell suspension	Foliar and ground application	Bactericide, fungicide	US	Anthracnose, bakanae, blights, molds, damping off diseases, melanose, mummy berry, phomopsis, pink root, post bloom fruit drop, powdery mildews, ramularia, rots, rusts, scabs, sclerotinia, shot hole, sigatoka, smut, spots, web blotch	Fruit blotch (*Actinovorax avenae*), blights, cankers, rots, specks and spots (*Erwinia* spp., *Pseudomonas* spp., *Xanthomonas* spp.), gumming disease (*Xanthomonas* spp.), olive knot (*Pseudomonas savastanoi*), pustule (*Xanthomonas spp.)*	Both: Tropical fruits, vegetables soybeans, sugarcanes, tree nuts, oilseed crops, olive, kiwifruit, F: Cereal grains, coffee, cotton, herbs and spices, grass seeds, grapes, nongrass animal feeds, peanut, pomegranate, tobacco	US:264-1152
				Chemigation, foliar and ground application		EU	Anthracnose, blights, blotches, clubroot, molds, damping off diseases, downy mildews, phomopsis, powdery mildews, rots, rusts, scabs, sclerotinia, sigatoga, spots	Blights, cankers, rots, specks and spots (*Erwinia* spp., *Pseudomonas* spp., *Xanthomonas* spp.)	Both: Citrus, pome and stone fruit trees, vegetables tropical fruits, pomegranate, Berries, F: herbs and spices, grapes, mushrooms, oilseed crops, ornamentals, tobacco, sugar beets	EU: Reg. (EU) 2015/1396Reg. (EU) 2020/421Reg. (EU) No 540/2011 (07/6/EC, Reg. (EU) 2019/168,Reg. (EU) 2018/524,Reg. (EU) No 487/2014)
	Serenade Max		Bacterial endospores (wettable powder)	Aerial and foliar application, chemigation ground incorporation	Bactericide, fungicide	US	Anthracnose, blights, blotches, molds, damping off diseases, downy mildews, eutypa, flyspeck, melanose, mummy berry, phomopsis, post bloom fruit drop, powdery mildews, rots, rusts, scabs, smuts, shot hole, spots	Fruit blotch (*Actinovorax avenae*), blights, cankers, rots, specks and spots (*Erwinia* spp., *Pseudomonas* spp., *Xanthomonas* spp.), gumming disease (*Xanthomonas* spp.), olive knot (*Pseudomonas savastanoi*), pustule (*Xanthomonas spp.)*	Both: Berries, cereal grains, pome and stone fruit trees, tree nuts, tropical fruits, vegetables F: citrus, grass seeds, grapes, nongrass animal feeds, oilseed crops, peanut	US: 264-1151
				Foliar application		EU	Blights, molds, rusts, scabs, sclerotinia	Blights, cankers, rots, specks and spots (*Erwinia* spp., *Pseudomonas* spp., *Xanthomonas* spp.), crown gall (*Agrobacterium tumefaciens*	Both: Berries, herbs and spices, grapes, pome and stone fruit trees, tobacco, tropical fruits, vegetables	Reg. (EU) 2015/1396Reg. (EU) 2020/421Reg. (EU) No 540/2011 (07/6/EC, Reg. (EU) 2019/168,Reg. (EU) 2018/524,Reg. (EU) No 487/2014)
	Serenade Opti		Bacterial endospores (wettable powder)	Aerial and ground application, chemigation	Bactericide, fungicide	US	Anthracnose, blights, blotches, molds, flyspeck, mummy berry, phomopsis, post bloom fruit drop, powdery mildews, rots, rusts, sclerotinia, shot hole, spots	Aerial Stem Rot (*Erwinia carotovora*), canker, (*Pseudomonas* spp.), fire blight (*Erwinia amylovora*), shot hole (*Xanthomonas pruni*), spots (*Pseudomonas* spp., *Xanthomonas* spp.),	Botth: Berries, pome and stone fruit trees, tree nuts, vegetables citrus, pomegranate, stevia F: Herbs and spices, grapes, kiwifruit, oilseed crops, peanut	US: 264-1160
	Serenade Soil		Concentrated cell suspension	Ground application	Bactericide, fungicide	US	damping off diseases, pink root, rots, sclerotinia	Rots (*Erwinia* spp.)	Both: Vegetables F: Berries, citrus fruit trees, peanut	US: 264-1152
	Jazz		Bacterial endospores (wettable powder)	Chemigation, ground incorporation	Fungicide	US	Molds		F: Mushrooms	US: 264-1151
*Bacillus mycoides* isolate J	Lifeguard	Certis USA L.L.C	Bacterial endospores (wettable powder)	Aerial and ground application, chemigation	Bactericide, fungicide	US	Anthracnose, blights, blotches, flyspeck, molds, downy mildews, mummy berry, powdery mildews, rusts, scabs, spots	Canker, spot and speck (*Pseudomonas* spp., *Xanthomonas* spp.), fire blight (*Erwinia amylovora)*	Both: Citrus and pome fruit trees, vegetables, grapes, peanuts, tobacco	US:70051-119
*Bacillus pumilus* QST 2808	Ballad Plus	Bayer Crop Science	Aqueous suspension of bacterial endospores, solids and solubles	Aerial and ground application, chemigation	Bactericide, fungicide	US	Blights, molds, downy mildews, powdery mildews, rots, rusts, smuts, spots, ramularia	Blights and speck (*Pseudomonas* spp., *Xanthomonas* spp.), pustule (*Xanthomonas* spp.)	Both: Cereal grains, oilseed crops, vegetables F: Grass grown for seed production, sugar beets	US:264-1153
	Bay 2000		Concentrated cell suspension	Seed application	Fungicide		Damping off diseases		F: Cereal grains, vegetables	US:264-118
	Sonata		Water suspension of bacterial endospores, solids and solubles	Aerial and ground application, chemigation	Fungicide		Blights, downy mildews, powdery mildews, rusts, scabs, spots		F: Berries, cereal grains, citrus, pome and stone fruit trees, herbs and spices, grapes, grass grown for seed production oilseed crops, roses, sugar beets, sweet corn, tree nuts, vegetables	US:264-1153
			Concentrated cell suspension	Foliar application	Fungicide	EU	Powdery mildews		F: Herbs and spices, grapes, seed production, tobacco, vegetables	EU: Reg. (EU) No 485/2014 (2011/253/EU)
*Pseudomonas fluorescens* A506	BlightBan A506	Nufarm Americas Inc.	Bacterial endospores (wettable powder)	Foliar application	Bactericide, fungicide	US	Molds	Fire blight (*Erwinia amylovora*), sour rot (*Acetobacter* spp.)	Both: Berries, pome and stone fruit trees, vegetables	US:228-710
*Sreptomyces lydicus* WYE 108	Actinovate AG	Novozymes	Bacterial endospores (wettable powder)	Chemigation, cutting/root, foliar, ground and seed application	Bactericide, fungicide	US	Damping off diseases, molds, rots	Bacterial spot (*Xanthomonas perforans*), walnut blight (*Xanthomonas arboricola* pv. *juglandis*)	Both: Stone fruit, vegetables F: Berries, cereal grains, citrus, pome fruit trees, herbs and spices, grapes, mushroom, oilseed crops, soybean, tree nuts, tropical fruits	US:73314-1
	Actinovate Lawn and Garden			Foliar, ground application	Bactericide, fungicide	US	Club root, damping off diseases, downy mildews, leaf curl, molds, patches, powdery mildews, rots, spots	Bacterial spot (*Xanthomonas perforans*), bacterial blast (*Pseudomonas syringae*), citrus canker (*Xanthomonas axopodis* pv. *citri*)	Both: Lawns, pome and stone fruit trees, ornamentals, vegetables	US:73314-1
				Foliar, ground application	Fungicide	EU	blights, damping off diseases, downy mildews, powdery mildews, rusts, spots		F: Lawns, ornamentals	Reg. (EU) No 917/2014 (Dossier complete (2011/253/EU))
	Actinovate SP			Cut/root, ground and seed application	Fungicide	US	Anthracnose, blights, damping off diseases, downy mildews, molds, patches, powdery mildews, rusts, spots		F: Lawns, ornamentals, vegetables	US:73314-1
	Actinovate STP			Dry seed application			Collapses, damping off diseases, rots, smuts		F: Cereal grains, cotton, herbs and spices, oilseed crops, peanut, sugar beets, vegetables	US:73314-4

## Data Availability

Not applicable.

## Supplementary Materials

The following are available online at https://www.mdpi.com/article/10.3390/biology10111202/s1, Video S1: Biofilm formation by *B. amyloliquefaciens* MBI600.
